# Curable Diseases

**Published:** 1875-11

**Authors:** E. H. Gibbs


### CURABLE DISEASES.

BY E. H. GIBBS, M.D.

It is my belief, confirmed by years of experience and observation, that nearly all diseases — not involving considerable change of structure — are curable. It is no exaggeration to say that a majority of persons, over forty years of age, are sufferers from some chronic affection of greater or less gravity,—the result, usually, of neglect or improper treatment. These cases are more numerous now than they were twenty years ago, and the reason is plain.

Formerly, the physician came to the bedside armed with the most powerful implements known to the healing art. He bled and purged and starved the patient till the enemy was routed, and if the patient survived, the disease did not. Then came the homœopathists, who seemed to go to the opposite extreme. They destroyed the old system of practice, but did not make converts of its votaries, who, from that time to this have been gravitating between the two extremes; and from their indecision and their fear of doing something that might not be regular, are responsible for a great deal of suffering. But the profession is becoming more liberal and more in sympathy with the people, and must, as a result of superior education and training, occupy its old position in the esteem and confidence of the public, to the exclusion of the ignorant and incompetent. The regular physician now employs remedies that he condemned even two years ago. There is nothing that he prizes so highly as "Electricity," and he gladly recognizes the merits of "Turkish baths" and the "Movement Cure." He might even prescribe sugar pills if he believed they were better than the more repulsive sort.

The knowledge of many of our best physicians seems to be limited to diagnoses of the cases that present themselves. They point out accurately the location and extent of a disease, but seem to have no thought of the treatment. Such as nature does not cure become chronic by neglect, and some become incurable.

I pin my faith to no single remedy. I believe that "Electricity" takes the lead in the treatment of all nervous diseases, rheumatism, tumors, and skin-diseases, but it must frequently be aided by other means. In the careful and skillful use of proper remedies success depends. With these, most diseases are remediable — many curable.

